# The impact of age on the implementation of evidence-based medications in patients with coronary artery disease and its prognostic significance: a retrospective cohort study

**DOI:** 10.1186/s12889-018-5049-x

**Published:** 2018-01-17

**Authors:** Tian-li Xia, Fang-yang Huang, Yi-ming Li, Hua Chai, Bao-tao Huang, Yuan-Wei-Xiang Ou, Qiao Li, Xiao-bo Pu, Zhi-liang Zuo, Yong Peng, Mao Chen, De-jia Huang

**Affiliations:** Department of Cardiology, West China Hospital, Sichuan University, 37 Guoxue Street, Chengdu, 610041 Chengdu, People’s Republic of China

**Keywords:** Coronary artery disease, Elderly, Evidence-based medications, Mortality

## Abstract

**Background:**

Elderly patients with coronary artery disease (CAD) frequently complicated with more cardiovascular risk factors, but received fewer evidence-based medications (EBMs). This study explored the association of EBMs compliance in different age groups and the risk of long-term death.

**Methods:**

A retrospective analysis was conducted from a single registered database. 2830 consecutive patients with CAD were enrolled and grouped into 3 categories by age. The primary end point was all-cause mortality and secondary endpoint is cardiovascular mortality.

**Results:**

The mean follow-up time was 30.25 ± 11.89 months and death occurred in 270 cases,including 150 cases of cardiac death. Cumulative survival curves indicated that the incidence rates of all-cause death and cardiovascular death increased with age (older than 75 years old vs. 60 to 75 years old vs. younger than 60 years old, mortality: 18.7% vs. 9.6% vs. 4.1%, *p* < 0.001; cardiovascular mortality: 10.3% vs. 5.1% vs. 2.7%, p < 0.001). The percentage of elderly patients using no EBMs was significantly higher than the percentages in the other age group (7.7% vs. 4.6% vs. 2.2%,*p* < 0.05). Cox regression analysis revealed the benefit of combination EBMs (all-cause mortality: hazard ratio [HR] 0.15, 95% CI 0.08–0.27; cardiac mortality: HR 0.08, 95% CI 0.04–0.19) for older CAD patients. Similar trends were found about different kinds of EBMs in elderly patients.

**Conclusions:**

Elderly patients with CAD had higher risk of death but a lower degree of compliance with EBMs usage. Elderly CAD patients could receive more clinical benefits by using EBMs.

**Electronic supplementary material:**

The online version of this article (10.1186/s12889-018-5049-x) contains supplementary material, which is available to authorized users.

## Background

After 30 years of rapid economic growth, China is facing the dual pressures of chronic non-communicable diseases and an ageing population [1, 2]. Elderly patients frequently have complications from various cardiovascular disease risk factors, such as hypertension, hyperlipidaemia, and diabetes, which are independently associated with cardiovascular events and deaths in patients with coronary artery disease (CAD) [3–5]. Studies have shown that CAD is the most common disease in people over 65 years of age and that 83% of patients who die of ischaemic heart disease are older than 65 years [3]. Current treatment for CAD have entered the era of evidence-based medications (EBMs), and the clinical diagnosis and treatment are implemented following the standard of care listed in the guidelines [6–9]. However, the current guidelines were established based on studies that did not take age factors into full account; in addition, many studies completely excluded elderly patients due to safety considerations [10–12]. Furthermore, when treating elderly patients, clinicians have more concerns for the therapeutic window and the possibility of side effects due to elderly patients’ unique physiological characteristics [13]. Therefore, current guidelines might have significant limitations regarding clinical compliance among elderly CAD patients. These limitations have not been adequately studied among CAD patients in China.

By analysing a single-centre cohort of 2830 CAD patients, this study explored the association of EBM compliance in CAD patients in different age groups and the risk of long-term death.

## Methods

### Study population

We investigated patients undergoing angiography from July 2008 to December 2010 included the West China Hospital CAD database. Patients met the inclusion criteria when they were restricted to participants with angiographic evidence of ≥50% stenosis in ≥1 coronary vessels. The exclusion criteria included pregnancy, malignancies, end stage renal disease (ESRD) with haemodialysis or renal transplant and severe liver or haematological diseases. Severe liver diseases were defined as liver insufficiency that lead to haemostatic disturbances, aminotransferases and bilirubin concentrations 3 times greater than the normal upper limits and other factors that may preclude angiography and subsequent EBM therapy [14]. Haematological diseases were defined as severe anaemia, myeloproliferative disorders, coagulopathies and haematological malignancies that may preclude angiography and subsequent EBM therapy. Patients with moderate and severe iron deficiency anaemia were excluded from our study. These inclusion and exclusion criteria were met by 3178 continuously enrolled CAD patients. A total of 302 patients were lost to follow-up and another 46 had incomplete follow-up data. There were 2830 patients included in the data analysis. The study protocol was approved by the local institutional review boards in accordance with the Declaration of Helsinki. Informed consent was obtained from all individual participants included in the study.

### Baseline characteristics

Demographic data, medical history, cardiovascular risk factors, vital signs at admission, medication at discharge, and final diagnosis were obtained from the patients’ electronic medical records and reviewed by a trained study coordinator. Blood sample were collected before angiography, and plasma biomarkers including markers of liver and kidney function (including the admission serum creatinine levels), blood glucose, serum lipids, and others, were analysed in the department of laboratory medicine, West China hospital, accredited by the College of American Pathologists. Hypertensive individuals were defined as those with systolic blood pressure (SBP) greater than 140 mmHg and/or diastolic blood pressure (DBP) greater than 90 mmHg and/or those receiving antihypertensive medications for 2 weeks. Diabetes was diagnosed when patients had previously undergone dietary treatment and/or had received additional oral antidiabetic or insulin medication or had a current fasting blood glucose level of ≥7.0 mmol/L or random blood glucose level ≥ 11.1 mmol/L. Dyslipidemia was defined as fasting serum total cholesterol (TC) level ≥ 5.18 mmol/L, and/or fasting serum low-density lipoprotein cholesterol (LDL-C) level ≥ 3.37 mmol/L, and/or fasting serum high-density lipoprotein-cholesterol (HDL-C) level of 1.04 mmol/L, and/or fasting serum triglycerides (TG) level ≥ 1.70 mmol/L, and/or those receiving treatment with drugs or therapeutic life-style change for dyslipidaemia. Acute myocardial infarction (AMI) was diagnosed by cardiologists, based on the triad of chest pain, electrocardiogram changes, and elevated cardiac troponin I levels (≥0.03 μg/L) or elevated cardiac troponin T levels (≥42 ng/L). Patients received care according to the usual practice; treatment was not affected by participation in this study.

### Follow-up and end points

The follow-up period ended in January 2013. Follow-up information was collected by contacting with patients, their family or physicians, follow-up information was collected. All data were corroborated with the hospital records. The primary end points in this study was all-cause mortality and the secondary endpoints is cardiovascular (CV) death. Death was considered cardiac-related when it was caused by AMI, significant arrhythmias, or refractory heart failure. Sudden unexpected death occurring without another explanation was also included.

### Statistical analyses

We conducted the post-hoc analysis on a retrospective basis. Baseline demographics and clinical characteristics were compared among patients categorized by age in 3 group corresponding to strata used to define elderly: Group 1: younger than 60 years old; Group 2: ranging from 60 to 75 years old and Group 3: older than 75 years old. The clinical characteristics of each of the three groups were presented as frequencies and percentages for categorical variables and as means ± standard deviation (SD) for continuous variables, respectively. Means were compared with analysis of variance (ANOVA) and nominal variables were compared with the chi-square test. The Kaplan-Meier and the log-rank test were used for survival analysis in relation to all-cause mortality and cardiovascular in CAD patients. The effects of discharged EBMs effects on all-cause mortality and mortality were considered in a Cox proportional hazards regression model in both unadjusted and adjusted fashion, and hazard ratios (HRs) were estimated with 95% confidence intervals (CIs). EBMs included aspirin, beta-receptor blockers, statins and angiotensin-converting enzyme inhibitors (ACEIs) or angiotensin-receptor blockers (ARBs) [8, 9]. Adjustments were made for the possible confounding effects of sex, history of hypertension, history of diabetes mellitus, history of heart failure, history of dyslipidaemia, smoking status, eGFR and hepatic enzymes. Because of the significant imbalances in baseline covariates between patients with and without EBMs, we used a 1:1 propensity score-matched pair method combined with covariate adjustment including sex, history of hypertension, history of diabetes mellitus, history of heart failure, history of dyslipidaemia, smoking status, eGFR and hepatic enzymes to analyse patients with and without the prescription of different kind of EBMs (for detail See Additional file 1). Then we constructed a Cox proportional hazards regression model in unadjusted fashion to compare outcomes among matched pairs of patients with and without the prescription of different kind. Increasingly adjusted models for composite effect of discharged medication on mortality were built for all-cause mortality and cardiovascular mortality to assess the three EBMs: beta-receptor blockers, statins and ACEIs or ARBs. Therefore, models were built as described below to classify the number of prescription regardless of the type of drugs: model 0: no medications; model 1: prescribed 1 type of EBM; model 2, prescribed 2 types of EBMs; and model 3, prescribed all 3 types of EBMs. Two-sided *p* values of less than 0.05 indicated statistical significance. All analyses were performed with SPSS software (version 19.0).

## Results

**Fig. 1:**
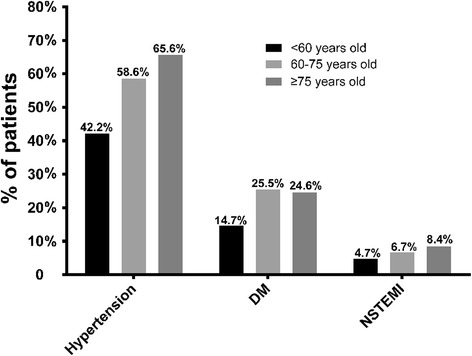
Discharge prescription of EBMs for CAD patients stratified by age (*P* < 0.05 for trend in EBMs, including aspirin, clopidogrel, dual antiplatelet, statins, beta-blockers, and ACEIs or ARBs, according to age). Abbreviations: EBMs, evidence-based medicines; CAD, coronary artery disease; ACEI, angiotensin-converting enzyme inhibitor; ARBs, angiotensin receptor blockers

A total of 2830 patients with CAD were included in the study. The the average age and its SD were 64.62 ± 10.54 years, and 20.3% of the patients were male. A total of 850 (30.0%) patients are younger than 60 years old, 1490 (52.6%) were in the range of 60 to 75 years old and 491(17.3%) were over 75 years old. The distribution of baseline data is shown in Table 1. The clinical features of the patients showed certain differences between groups. Older patients have a lower proportion of dyslipidaemia, and higher proportion of hypertension, diabetes mellitus and heart failure (*p* < 0.001). The percentage of patients diagnosed with non-ST segment elevation myocardial infarction (NSTEMI) had a higher trending proportion (Fig. 1).

**Table 1:** Baseline characteristics of the study population

Characteristic of patients		Age			
	Total *N* = 2830	< 60 years old *N* = 850	60–75 years old *N* = 1489	≤75 years old *N* = 491	p
Age, yrs	64.62 ± 10.54	51.63 ± 6.37	67.48 ± 4.23	78.42 ± 2.93	< 0.001
Gender, men, *n* (%)	575(20.32)	83(9.76)	370(24.85)	122(24.85)	< 0.001
BMI, Kg/m^2^	24.20 ± 3.21	24.60 ± 2.95	24.12 ± 0.08	23.70 ± 0.17	< 0.001
Smoking, *n* (%)	888(33.40)	331(40.91)	434(31.31)	123(26.51)	< 0.001
Drinking, *n* (%)	647(24.80)	237(30.15)	320(23.49)	90(19.52)	< 0.001
Medical history					
Pre-hypertension, *n* (%)	1546(54.86)	357(42.20)	870(58.55)	319(65.64)	< 0.001
Hyperlipidemia, *n* (%)	474(16.83)	169(19.98)	257(17.32)	48(9.86)	< 0.001
Pre-diabetes mellitus, *n* (%)	622(22.08)	124(14.66)	378(25.47)	120(24.64)	< 0.001
Laboratory values					
LVEF, %	52.63 ± 23.07	51.26 ± 23.46	54.08 ± 22.01	50.49 ± 25.27	< 0.001
eGFR, ml/min/1.73m^2^	80.39 ± 40.97	93.49 ± 63.63	76.29 ± 23.84	70.11 ± 22.25	< 0.001
Total cholesterol, mmol/L	4.09 ± 1.14	4.21 ± 1.29	4.04 ± 1.09	4.02 ± 0.97	< 0.001
LDL_C, mmol/L	2.40 ± 0.94	2.52 ± 1.06	2.36 ± 0.90	2.31 ± 0.81	< 0.001
Blood glucose, mmol/L	7.03 ± 3.22	6.8 ± 3.04	7.05 ± 3.39	7.37 ± 2.98	0.010
Severity of CAD					
Number of stents, n	1.87 ± 1.11	1.77 ± 1.07	1.88 ± 1.11	2.05 ± 1.14	< 0.001
Diagnose					
Unstable angina, *n* (%)	1495(52.83)	422(49.65)	831(55.81)	242(49.29)	< 0.001
STEMI, *n* (%)	392(13.85)	139(16.35)	179(12.02)	74(15.07)	0.010
NSTEMI, *n* (%)	180(6.36)	40(4.71)	99(6.65)	41(8.35)	0.030
All death, *n* (%)	270(9.54)	35(4.12)	143(9.60)	92(18.74)	< 0.001
CV death, *n* (%)	150(5.30)	23(2.71)	76(5.10)	51(10.39)	< 0.001
Discharge medications					
Aspirin, *n* (%)	2608(92.16)	807(94.94)	1374(92.28)	427(86.97)	< 0.001
Clopidogrel, *n* (%)	2522(89.12)	776(91.29)	1332(89.46)	414(84.32)	0.006
Dual-antiplatelet, *n* (%)	2446(86.43)	761(89.53)	1288(86.50)	397(80.86)	< 0.001
Statin, *n* (%)	2521(89.08)	778(91.53)	1307(87.78)	436(88.80)	0.023
CCB, *n* (%)	744(26.29)	162(19.06)	433(29.08)	149(30.35)	< 0.001
ACE inhibitors or ARBs, *n* (%)	1603(56.64)	436(51.29)	880(59.10)	287(58.45)	< 0.001
Beta-receptor blockers, *nw* (%)	1874(66.22)	605(71.18)	990(66.49)	279(56.82)	< 0.001

Data are expressed as means± SD or counts and percentages, as appropriate. Abbreviations: BMI: body mass index, DM: diabetes mellitus, LVEF: left ventricular ejection fraction, eGFR: estimated glomerular filtration rate, LDL-C: low-density lipoprotein-cholesterol, STEMI: ST-segment elevated myocardial infarction, NSTEMI: non-ST-segment elevated myocardial infarction, SD: standard deviation. ACE, angiotensin-converting enzyme; ARBs, angiotensin-receptor blockers

**Table 2:** Multivariate Cox’s proportional hazards regression model each evidence-based medications

		< 60 years old			60–75 years old			≥75 years old		
Medicines	Mortality	Unadjusted HR (95% CI)	Adjusted HR (98% CI)	Propensity score analysis unadjusted HR (98% CI)	Unadjusted HR (96% CI)	Adjusted HR (99% CI)	Propensity score analysis unadjusted HR (98% CI)	Unadjusted HR (97% CI)	Adjusted HR (100% CI)	Propensity score analysis unadjusted HR (98% CI)
Aspirin	All-cause death	0.13 (0.06–0.27)	0.12 (0.05–0.25)	0.19 (0.04–0.86)	0.16 (0.11–0.22)	0.17 (0.11–0.24)	0.17 (0.08–0.36)	0.21 (0.13–0.32)	0.22 (0.14–0.34)	0.21 (0.09–0.45)
	CV death	0.07 (0.03–0.15)	0.06 (0.02–0.15)	0.09 (0.01–0.74)	0.11 (0.07–0.18)	0.12 (0.07–0.19)	0.16 (0.06–0.41)	0.17 (0.09–0.30)	0.17 (0.09–0.31)	0.26 (0.10–0.66)
Clopidogrel	All-cause death	0.16 (0.08–0.32)	0.14 (0.07–0.28)	0.26 (0.07–0.94)	0.25 (0.18–0.36)	0.26 (0.18–0.38)	0.20 (0.10–0.40)	0.30 (0.19–0.47)	0.30 (0.19–0.47)	0.42 (0.23–0.77)
	CV death	0.11 (0.05–0.27)	0.11 (0.05–0.27)	0.11 (0.01–0.85)	0.14 (0.09–0.22)	0.15 (0.09–0.23)	0.14 (0.05–0.36)	0.27 (0.15–0.48)	0.26 (0.14–0.48)	0.42 (0.19–0.95)
Dual-antiplatelet	All-cause death	0.18 (0.09–0.36)	0.15 (0.07–0.30)	0.06 (0.01–0.47)	0.26 (0.18–0.37)	0.27 (0.19–0.38)	0.18 (0.09–0.36)	0.33 (0.21–0.51)	0.34 (0.22–0.53)	0.38 (0.20–0.69)
	CV death	0.12 (0.05–0.29)	0.12 (0.05–0.28)	0.01 (0–1.53)	0.17 (0.11–0.27)	0.17 (0.11–0.28)	0.16 (0.07–0.38)	0.31 (0.17–0.55)	0.31 (0.17–0.56)	0.42 (0.19–0.92)
Statins	All-cause death	0.18 (0.08–0.38)	0.16 (0.08–0.35)	0.43 (0.15–1.24)	0.29 (0.20–0.41)	0.30 (0.21–0.43)	0.08 (0.03–0.23)	0.20 (0.13–0.31)	0.20 (0.12–0.31)	0.31 (0.15–0.61)
	CV death	0.10 (0.04–0.25)	0.10 (0.04–0.24)	0.29 (0.08–1.05)	0.22 (0.14–0.35)	0.23 (0.14–0.37)	0.10 (0.03–0.32)	0.15 (0.09–0.28)	0.15 (0.08–0.27)	0.31 (0.13–0.75)
ACEIs or ARBs	All-cause death	0.86 (0.43–1.74)	0.89 (0.44–1.80)	0.83 (0.42–1.64)	0.48 (0.34–0.68)	0.50 (0.35–0.70)	0.43 (0.29–0.64)	0.48 (0.31–0.74)	0.54 (0.35–0.84)	0.44 (0.27–0.70)
	CV death	0.54 (0.22–1.35)	0.54 (0.22–1.34)	0.47 (0.19–1.14)	0.41 (0.25–0.65)	0.42 (0.26–0.68)	0.31 (0.17–0.55)	0.41 (0.23–0.73)	0.45 (0.25–0.83)	0.35 (0.18–0.69)
Beta-blockers	All-cause death	0.49 (0.25–0.97)	0.50 (0.25–1.00)	0.60 (0.27–1.32)	0.34 (0.24–0.47)	0.35 (0.25–0.48)	0.36 (0.24–0.55)	0.65 (0.43–0.99)	0.65 (0.42–1.00)	0.63 (0.40–0.99)
	CV death	0.33 (0.14–0.76)	0.35 (0.15–0.83)	0.37 (0.13–1.03)	0.31 (0.19–0.48)	0.32 (0.20–0.51)	0.27 (0.14–0.50)	0.44 (0.25–0.78)	0.44 (0.25–0.80)	0.34 (0.17–0.67)

*Abbreviations*: *CAD* Coronary artery disease, *CI* Confidence interval, *CV* Death: cardiovascular death, *HR* Hazard ratio, *LDL-C* Low-density lipoprotein-cholesterol, STEMI: ST-segment elevated myocardial infarction.Adjusted factor: sex, history of hypertension, history of diabetes mellitus, and history of heart failure, history of dyslipidemia, smoking status, eGFR and hepatic enzymes

**Fig. 2:**
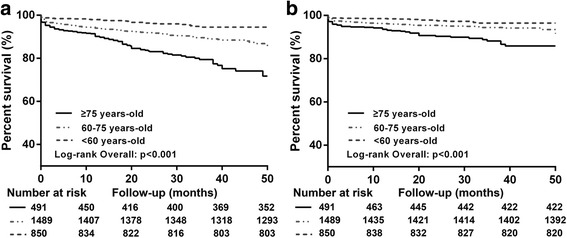
Kaplan-Meier estimates of time to all-cause death (**a**) and to cardiovascular death (**b**) according to age at baseline

The average follow up was 30.25 ± 11.89 months. A total of 270 cases of all-cause death occurred (mortality, 9.5%, 95% CI: 8.5%–10.7%), including 150 cases of cardiovascular death (cardiovascular mortality, 5.3%, 95% CI, 4.5%–6.2%). The survival curve of patients older than 75 years old had a significantly higher risk of all-cause death (panel A) and cardiovascular death (panel B) than the group ranging from 60 to 75 years old and the group younger than 60 years old (mortality, Group 3 vs. Group 1: 18.7% vs. 4.1%, *p* < 0.001; Group 2 vs. Group 1: 9.6% vs. 4.1%, *p* < 0.001; cardiovascular mortality, Group 3 vs. Group 1: 10.3% vs. 2.7%, *p* < 0.001; Group 2 vs. Group 1: 5.1% vs. 2.7%, *p* < 0.001) (Fig. 2).

**Fig. 3:**
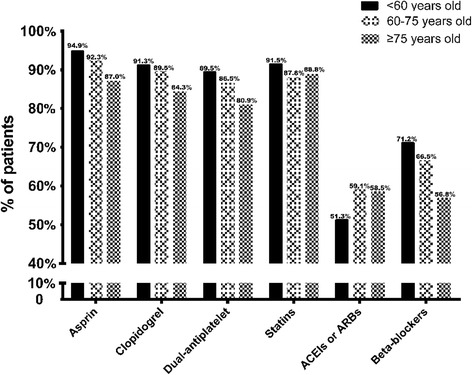
Several clinical characteristics of CAD patients stratified by age. (all *p*_for trend_ < 0.05 in hypertension, DM and NSTEMI according to age). Abbreviations: DM, diabetes mellitus; NSTEMI, non-ST-elevation myocardial infarction

**Fig. 4:**
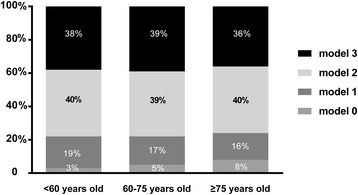
Relative portion of types of prescription of EBMs on discharge in different age groups. Abbreviations: EBMs, evidence-based medicines

Approximately 92.2% of CAD patients were discharged with aspirin, 86.4% with aspirin and clopidogrel, 66.22% with beta-blockers, 56.6% with ACE inhibitors, 89.1% with statins, and 36% with the combination of aspirin, clopidogrel, beta-blockers, ACE inhibitors, and statins. The medicine proportion of ere different in all groups. In the group 2 and 3, the proportion of EBMs prescribed at discharge is significantly lower than the younger but ACEIs or ARBs is opposite. (asprin, dual-antiplatelet, and beta-blockers: all *p* for tends < 0.001; and statins: *p* = 0.023) (Fig. 3). Meanwhile, we analysed the combined use of beta-blockers, statins, and ACEIs or ARBs among the EBMs (Fig. 4). The percentage of patients over 75 years old using no EBMs was significantly higher than the percentages in the other 2 groups (7.7% vs. 4.6% vs. 2.2%,*p* for trend < 0.05) (Fig. 4).

In multivariate Cox’s proportional hazards regression model analyses each kind of EBMs (beta-blockers, statins, and ACEIs or ARBs) alone was considered an important modifiable protective factor of both all-cause death and CV death in patients ranged from 60 to 75 years old or over 75 years old (Table 2). For ACEI or ARB use, Group 3 and 2 benefit more than Group 1 regarding to all-cause death and CV death (all-cause death: Group 1: HR = 0.89, 95% CI, 0.44–1.80; Group 2: HR =0.48, 95% CI, 0.34–0.68; Group 3: HR =0.54, 95% CI, 0.35–0.84; CV death: Group 1: HR = 0.54, 95% CI, 0.22–1.34; Group 2: HR =0.42, 95% CI, 0.26–0.68; Group 3: HR =0.45, 95% CI, 0.25–0.83). In the unadjusted Cox’s proportional hazards regression model analysis of the patients after propensity score matching, we obtained constant results with the adjusted analysis of patients before propensity score matching (Table 2). The combination of EBM use resulted in a greater reduction in the risk of all-cause death even in patients older than 75 years. Meanwhile, similar results were observed in the analysis of CV death (Table 3).

**Table 3:** Multivariate Cox’s proportional hazards regression model on combination therapy of EBMs

Medication	Age (years-old)		
Combinations	< 60	60–75	≥75
All-cause death			
Model 0	1.00	1.00	1.00
Model 1	0.11 (0.03–0.34)	0.15 (0.09–0.26)	0.17 (0.08–0.33)
Model 2	0.06 (0.02–0.18)	0.12 (0.08–0.19)	0.15 (0.08–0.26)
Model 3	0.10 (0.04–0.25)	0.08 (0.05–0.13)	0.15 (0.08–0.27)
CV death			
Model 0	1.00	1.00	1.00
Model 1	0.07 (0.02–0.27)	0.10 (0.05–0.20)	0.10 (0.04–0.26)
Model 2	0.04 (0.01–0.13)	0.07 (0.04–0.13)	0.10 (0.05–0.22)
Model 3	0.04 (0.01–0.14)	0.07 (0.04–0.12)	0.08 (0.04–0.19)

Adjusted factor: sex, history of hypertension, history of diabetes mellitus, and history of heart failure, history of dyslipidemia, smoking status, eGFR and hepatic enzymes. Model 0: no medication; model 1: prescribed 1 type of EBMs; model 2, prescribed 2 types of EBMs; model 3, prescribed all 3 types of EBMs. Three types of EBMs included: statin, beta-blockers, and RAAS inhibitors (ACEIs or ARBs). Abbreviations: EBMs: evidence-based medications, CAD: coronary artery disease, CI: confidence interval, CV death: cardiovascular death, HR: hazard ratio

## Discussion

This study found that elderly patients had complications with more cardiovascular disease risk factors and had higher all-cause death and cardiovascular events. However, the proportion of elderly patients receiving EBMs was lower than that of younger patients. Patients who had higher compliance with EBMs exhibited a lower risk of death.

Age is an important risk factor for poor prognosis in patients with CAD [15]. On one hand, with increases in age and organ ageing, the capacity to survive cardiovascular events declines; on the other hand, due to concerns regarding drug tolerance and side effects, elderly patients often receive insufficiently effective drugs, resulting in poor prognosis [16–19]. Previous studies have shown that complications or dysfunctions of liver and kidney limit the use of statins and ACEI/ ARB drugs [17, 18]. Concerns regarding the risk of bleeding and gastrointestinal discomfort limit the use of aspirin in elderly patients [20, 21]. Similar with previous foreign reports [3, 22, 23], our study suggests that EBM compliance among Chinese patients with CAD is notably inadequate.

The reasons for inadequate compliance with EBMs for elderly patients are not clearly elucidated. Based on previous studies, we speculate that the inadequate compliance might be caused by the following reasons. (1) Due to organ dysfunction, elderly patients had concerns about drug side effects. For example, due to high risk of cerebral haemorrhage and gastrointestinal bleeding, elderly patients had more concerns about the risk of bleeding associated with anti-platelet drugs, especially double antiplatelet therapy [21, 24]; due to liver and renal dysfunction, elderly patients had major concerns about statin-induced liver injury and ACEI/ARB-induced renal damage [25–27]; and due to conduction dysfunction and high incidence of chronic obstructive pulmonary disease (COPD), elderly patients had concerns that a beta-blocker would aggravate slow arrhythmia and wheezing [28, 29]. (2) Elderly patients had poor tolerance for drugs. Elderly patients had a narrow therapeutic window, with the minimum effective concentration being very close to the minimum toxic concentration [30]. (3) The physical conditions of elderly patients is frequently changing. CAD is a chronic disease, and the long-term compliance with EBMs requires complicated medication dose adjustment and maintenance [13]. (4) Elderly patients often suffered from multiple diseases and therefore bear high medication costs [31]. (5) Elderly patients had decreased memory and limited social support, negatively affecting long-term medication compliance [32]. The above factors not only led to low EBM compliance among the elderly patients but also discouraged physicians from prescribing EBMs to elderly CAD patients. However, the available evidence does not show that elderly CAD patients should reduce the use of EBMs [33–35]. Two meta-analyses showed that statin use in elderly patients over 65 years of age was able to reduce the risk of all-cause mortality by 22%, coronary-related deaths by 30%, myocardial infarction by 26% and revascularization by 30% [36, 37]. Another meta-analysis showed that the use of an ACEI or ARB in elderly patients with cardiovascular disease may increase the risk of vascular oedema, hypotension, and renal impairment, but cautious use could still reduce the risk of all-cause death, cardiac death, myocardial infarction, heart failure, and other clinical events [38]. Soumerai et al. found that the risk of death was 14% lower in elderly patients with acute myocardial infarction who were treated with beta-blockers at discharge, which was consistent with the results of randomized controlled trials of young and low-risk populations [39]. The results of the present study also showed that a higher degree of EBM compliance corresponded to better prognosis, suggesting that elderly CAD patients should also adhere to the guidelines and EBMs.

Several limitations of this study should be addressed. First, the registry made it difficult to completely avoid selection bias and confounding factors including information of activity of daily livings. In current study, the prescription rate of ACEI/ARBs was higher in those aged 60–75 and > 75 than those aged < 60, which was a converse trend to other EBMs. The possible explain was that ACEI/ARBs was not only an EBMs for ACS, but also used as an antihypertensive agents. Elderly CAD patients were complicated more hypertension than younger patients, and had more chance to take ACEI/ARBs. After excluding the patients with hypertension, the prescription rate of ACEI/ARBs were equal in three groups of different ages. (see Additional files 2) Second, the samples in this single-centre study were subject to geographical restrictions, which affected their representativeness and generalization. Third, patients older than 80 years old are thought to be more insufficient in EBM use than younger patients [40, 41]. We conducted analysis on patients older than 80 but the group sizes (*n* = 155) was too small to obtain an adequate statistical power. (see Additional files 3 and 4) Finally, this study focused on four types of medications, antiplatelet drugs, statin, beta-blocker and ACEI/ARB. The effects of other medications were not studied. In conclusion, caution must be taken when analysing the results of this study. However, attention should be paid to the insufficient use of EBMs and its potential impact on prognosis in elderly patients with CAD. High-quality research reports are needed to provide more clinical evidence and experience in the use of EBMs in elderly patients with CAD.

## Conclusions

Elderly patients with CAD had higher risk of cardiovascular disease but had a lower degree of compliance with EBMs usage to prevent cardiovascular events. Elderly CAD patients, similar to younger CAD patients, could also receive more clinical benefits by strictly adhering to the use of EBMs. Therefore, EBMs should also be actively promoted for elderly CAD patients.

## Additional files

Additional file 1: Number of matched pairs, before-matched and c-statistic. (DOCX 16 kb)

Additional file 2: Discharge prescription of ACEI/ARBs for CAD patients (panel A) and CAD patients without hypertension (panel B) stratified by age. Abbreviations: CAD, coronary artery disease; ACEI, angiotensin-converting enzyme inhibitor; ARBs, angiotensin receptor blockers. (DOCX 95 kb)

Additional file 3: Multivariate Cox’s proportional hazards regression model each evidence-based medications. Abbreviations: CAD: coronary artery disease, CI: confidence interval, CV death: cardiovascular death, HR: hazard ratio, LDL-C: low-density lipoprotein-cholesterol, STEMI: ST-segment elevated myocardial infarction. Adjusted factor: sex, history of hypertension, history of diabetes mellitus, and history of heart failure, history of dyslipidemia, smoking status, eGFR and hepatic enzymes. (DOCX 13 kb)

Additional file 4: Multivariate Cox’s proportional hazards regression model on combination therapy of EBMs. Adjusted factor: sex, history of hypertension, history of diabetes mellitus, and history of heart failure, history of dyslipidemia, smoking status, eGFR and hepatic enzymes. Model 0: no medication; model 1: prescribed 1 type of EBMs; model 2, prescribed 2 types of EBMs; model 3, prescribed all 3 types of EBMs. Three types of EBMs included: statin, beta-blockers, and RAAS inhibitors (ACEIs or ARBs). Abbreviations: EBMs: evidence-based medications, CAD: coronary artery disease, CI: confidence interval, CV death: cardiovascular death, HR: hazard ratio. (DOCX 13 kb)

## Abbreviations

ACEI: Angiotensin-converting enzyme inhibitors
AMI: Acute myocardial infarction
ANOVA: Analysis of variance
ARB: Angiotensin-receptor blocker
CAD: Coronary artery disease
CI: Confidence interval
COPD: Chronic obstructive pulmonary disease
CV death: Cardiovascular death
DBP: Diastolic blood pressure
EBMs: Evidence-based medications
ESRD: End stage renal disease
HDL-C: High-density lipoprotein-cholesterol
HR: Hazard ratio
LDL-C: Low-density lipoprotein cholesterol
NSTEMI: Non-ST segment elevation myocardial infarction
SBP: Systolic blood pressure
TC: Total cholesterol
TG: Triglycerides
