# Supplementary material for: The impact of age on the implementation of evidence-based medications in patients with coronary artery disease and its prognostic significance: a retrospective cohort study
Source: BMC Public Health. 2018 Jan 17;18:150. doi: 10.1186/s12889-018-5049-x (PMC5772723; doi:10.1186/s12889-018-5049-x)
Supplement: Supplementary file 1 — Number of matched pairs, before-matched and c-statistic. (DOCX 16 kb) [file 12889_2018_5049_MOESM1_ESM.docx]

Additional file 1. Number of matched pairs, before-matched and c-statistic.

|  |  | After-match | | Before-match | | c-statistic |
| --- | --- | --- | --- | --- | --- | --- |
|  |  | Control | Treated | Control | Treated |  |
| <60 years old | Aspirin | 72 | 72 | 72 | 778 | 0.854 |
|  | Clopidogrel | 245 | 245 | 245 | 605 | 0.789 |
|  | Statins | 414 | 414 | 414 | 436 | 0.958 |
|  | ACEIs or ARBs | 42 | 41 | 43 | 807 | 0.903 |
|  | Beta-blockers | 71 | 71 | 74 | 776 | 0.898 |
| 60-75 years old | Aspirin | 115 | 115 | 115 | 1374 | 0.965 |
|  | Clopidogrel | 499 | 499 | 499 | 990 | 0.930 |
|  | Statins | 182 | 182 | 182 | 1307 | 0.962 |
|  | ACEIs or ARBs | 609 | 609 | 609 | 880 | 0.895 |
|  | Beta-blockers | 157 | 157 | 157 | 1332 | 0.834 |
| ≤75 years old | Aspirin | 77 | 77 | 77 | 414 | 0.922 |
|  | Clopidogrel | 64 | 64 | 64 | 427 | 0.909 |
|  | Statins | 204 | 204 | 204 | 287 | 0.927 |
|  | ACEIs or ARBs | 55 | 55 | 55 | 436 | 0.843 |
|  | Beta-blockers | 212 | 212 | 212 | 279 | 0.651 |
