# Supplementary material for: The impact of age on the implementation of evidence-based medications in patients with coronary artery disease and its prognostic significance: a retrospective cohort study
Source: BMC Public Health. 2018 Jan 17;18:150. doi: 10.1186/s12889-018-5049-x (PMC5772723; doi:10.1186/s12889-018-5049-x)
Supplement: Supplementary file 2 — Discharge prescription of ACEI/ARBs for CAD patients (panel A) and CAD patients without hypertension (panel B) stratified by age. Abbreviations: CAD, coronary artery disease; ACEI, angiotensin-converting enzyme inhibitor; ARBs, angiotensin receptor blockers. (DOCX 95 kb) [file 12889_2018_5049_MOESM2_ESM.docx]

Additional file 2. Discharge prescription of ACEI/ARBs for CAD patients (panel A) and CAD patients without hypertension (panel B) stratified by age.





Abbreviations: CAD, coronary artery disease; ACEI, angiotensin-converting enzyme inhibitor; ARBs, angiotensin receptor blockers.
