# Supplementary material for: The impact of age on the implementation of evidence-based medications in patients with coronary artery disease and its prognostic significance: a retrospective cohort study
Source: BMC Public Health. 2018 Jan 17;18:150. doi: 10.1186/s12889-018-5049-x (PMC5772723; doi:10.1186/s12889-018-5049-x)
Supplement: Supplementary file 3 — Multivariate Cox’s proportional hazards regression model each evidence-based medications. Abbreviations: CAD: coronary artery disease, CI: confidence interval, CV death: cardiovascular death, HR: hazard ratio, LDL-C: low-density lipoprotein-cholesterol, STEMI: ST-segment elevated myocardial infarction. Adjusted factor: sex, history of hypertension, history of diabetes mellitus, and history of heart failure, history of dyslipidemia, smoking status, eGFR and hepatic enzymes. (DOCX 13 kb) [file 12889_2018_5049_MOESM3_ESM.docx]

Additional file 3. Multivariate Cox's proportional hazards regression model each evidence-based medications

|  |  | <65 years old | | 65-80 years old | | ≥80 years old | |
| --- | --- | --- | --- | --- | --- | --- | --- |
| Medicines | Mortality | Unadjusted HR (95%CI) | Adjusted HR (95%CI) | Unadjusted HR (95%CI) | Adjusted HR (95%CI) | Unadjusted HR (95%CI) | Adjusted HR (95%CI) |
| Aspirin | All-cause death | 0.10 (0.06-0.17) | 0.10 (0.06-0.19) | 0.18 (0.13-0.25) | 0.18 (0.13-0.25) | 0.28 (0.13-0.60) | 0.25 (0.10-0.61) |
|  | CV death | 0.05 (0.03-0.10) | 0.06 (0.03-0.12) | 0.15 (0.10-0.23) | 0.14 (0.09-0.22) | 0.23 (0.08-0.62) | 0.19 (0.06-0.67) |
| Clopidogrel | All-cause death | 0.16 (0.09-0.27) | 0.17 (0.09-0.30) | 0.26 (0.19-0.35) | 0.26 (0.19-0.36) | 0.35 (0.17-0.75) | 0.32 (0.14-0.74) |
|  | CV death | 0.10 (0.05-0.19) | 0.11 (0.06-0.23) | 0.18 (0.12-0.27) | 0.18 (0.12-0.28) | 0.31 (0.11-0.83) | 0.29 (0.09-0.91) |
| Dual-antiplatelet | All-cause death | 0.16 (0.09-0.27) | 0.17 (0.10-0.30) | 0.27 (0.20-0.36) | 0.27 (0.20-0.37) | 0.51 (0.24-1.08) | 0.47 (0.20-1.12) |
|  | CV death | 0.09 (0.05-0.18) | 0.11 (0.05-0.22) | 0.22 (0.15-0.34) | 0.22 (0.15-0.34) | 0.41 (0.15-1.12) | 0.38 (0.12-1.23) |
| Statins | All-cause death | 0.21 (0.12-0.38) | 0.24 (0.13-0.43) | 0.24 (0.18-0.33) | 0.24 (0.17-0.33) | 0.23 (0.11-0.50) | 0.21 (0.09-0.48) |
|  | CV death | 0.16 (0.08-0.32) | 0.20 (0.10-0.41) | 0.18 (0.12-0.27) | 0.18 (0.12-0.27) | 0.21 (0.08-0.56) | 0.20 (0.06-0.62) |
| ACEIs or ARBs | All-cause death | 0.67 (0.39-1.15) | 0.63 (0.36-1.10) | 0.50 (0.37-0.67) | 0.54 (0.40-0.73) | 0.44 (0.21-0.90) | 0.55 (0.25-1.21) |
|  | CV death | 0.44 (0.22-0.88) | 0.44 (0.21-0.92) | 0.41 (0.28-0.62) | 0.42 (0.27-0.64) | 0.50 (0.19-1.32) | 0.66 (0.22-1.94) |
| Beta-blockers | All-cause death | 0.44 (0.26-0.76) | 0.45 (0.26-0.78) | 0.41 (0.31-0.55) | 0.43 (0.32-0.58) | 0.58 (0.29-1.16) | 0.58 (0.28-1.22) |
|  | CV death | 0.35 (0.18-0.68) | 0.37 (0.19-0.75) | 0.33 (0.22-0.49) | 0.34 (0.22-0.51) | 0.37 (0.14-1.00) | 0.38 (0.13-1.11) |

Abbreviations: CAD: coronary artery disease, CI: confidence interval, CV death: cardiovascular death, HR: hazard ratio, LDL-C: low-density lipoprotein-cholesterol, STEMI: ST-segment elevated myocardial infarction.
Adjusted factor: sex, history of hypertension, history of diabetes mellitus, and history of heart failure, history of dyslipidemia, smoking status, eGFR and hepatic enzymes
