# Supplementary material for: The impact of age on the implementation of evidence-based medications in patients with coronary artery disease and its prognostic significance: a retrospective cohort study
Source: BMC Public Health. 2018 Jan 17;18:150. doi: 10.1186/s12889-018-5049-x (PMC5772723; doi:10.1186/s12889-018-5049-x)
Supplement: Supplementary file 4 — Multivariate Cox’s proportional hazards regression model on combination therapy of EBMs. Adjusted factor: sex, history of hypertension, history of diabetes mellitus, and history of heart failure, history of dyslipidemia, smoking status, eGFR and hepatic enzymes. Model 0: no medication; model 1: prescribed 1 type of EBMs; model 2, prescribed 2 types of EBMs; model 3, prescribed all 3 types of EBMs. Three types of EBMs included: statin, beta-blockers, and RAAS inhibitors (ACEIs or ARBs). Abbreviations: EBMs: evidence-based medications, CAD: coronary artery disease, CI: confidence interval, CV death: cardiovascular death, HR: hazard ratio. (DOCX 13 kb) [file 12889_2018_5049_MOESM4_ESM.docx]

Additional file 4. Multivariate Cox's proportional hazards regression model on combination therapy of EBMs

| Medication | Age(years-old) | | |
| --- | --- | --- | --- |
| Combinations | <65 | 65-80 | ≥80 |
| All-cause death |  |  |  |
| Model 0 | 1 | 1 | 1 |
| Model 1 | 0.08 (0.03-0.19) | 0.14 (0.09-0.23) | 0.25 (0.07-0.83) |
| Model 2 | 0.06 (0.03-0.13) | 0.13 (0.09-0.19) | 0.09 (0.03-0.33) |
| Model 3 | 0.07 (0.03-0.15) | 0.09 (0.06-0.14) | 0.19 (0.07-0.52) |
| CV death |  |  |  |
| Model 0 | 1 | 1 | 1 |
| Model 1 | 0.05 (0.02-0.16) | 0.09 (0.05-0.18) | 0.23 (0.05-1.10) |
| Model 2 | 0.05 (0.02-0.12) | 0.08 (0.05-0.13) | 0.03 (0.00-0.32) |
| Model 3 | 0.05 (0.02-0.12) | 0.06 (0.03-0.1) | 0.19 (0.05-0.71) |

Adjusted factor: sex, history of hypertension, history of diabetes mellitus, and history of heart failure, history of dyslipidemia, smoking status, eGFR and hepatic enzymes. Model 0: no medication; model 1: prescribed 1 type of EBMs; model 2, prescribed 2 types of EBMs; model 3, prescribed all 3 types of EBMs. Three types of EBMs included: statin, beta-blockers, and RAAS inhibitors (ACEIs or ARBs).
Abbreviations: EBMs: evidence-based medications, CAD: coronary artery disease, CI: confidence interval, CV death: cardiovascular death, HR: hazard ratio.
